# Fusion rate of 89% after knee arthrodesis using an intramedullary nail: a mono-centric retrospective review of 48 cases

**DOI:** 10.1007/s00167-021-06693-7

**Published:** 2021-08-29

**Authors:** Anaïs Luyet, Sylvain Steinmetz, Nicolas Gallusser, David Roche, Arnaud Fischbacher, Christophe Tissot, Olivier Borens

**Affiliations:** 1 Department of Orthopaedics and Traumatology, Lausanne University Hospital, University of Lausanne, Rue du Bugnon 46, 1011 Lausanne, Switzerland; 2 Clinique de la Source, Avenue Bergières 2, 1004 Lausanne, Switzerland

**Keywords:** Knee arthrodesis, Intramedullary nail, Total knee arthroplasty infection, Wichita

## Abstract

**Purpose:**

Knee arthrodesis is an established procedure for limb salvage in cases of recurrent infection, total knee arthroplasty soft tissue defect, poor bone stock or a deficient extensor mechanism. Surgical options include compression plate, external fixator and arthrodesis nail. Different types of nail exist: long fusion nail, short modular nail and bridging nail. This study presents the results on knee arthrodesis using different types of intramedullary nails. The aim is to assess if a specific type of nail has a better fusion rate, clinical outcome and lower complication rate.

**Methods:**

A mono-centric retrospective study of 48 knees arthrodesis was performed between 2000 and 2018. 15 T2^™^ Arthrodesis Nail, 6 OsteoBridge^®^ Knee Arthrodesis and 27 Wichita^®^ fusion nail were used. The mean clinic and radiological follow-up was 9.8 ± 3.8 years (2.6–18 years).

**Results:**

Fusion rate was 89.6%. Time to fusion was 6.9 months. Mean Parker score was 6.9/9 points. Visual Analogic Scale was 1.9. The Wichita^®^ fusion nail showed better results in terms of fusion, time to fusion and clinical outcome measured by Parker score and VAS but without statistical significance. The early revision rate was 10.4% and 20.8% presented a late complication requiring a surgery, due to nonunion or infection. 93.3% of infection was cured. Two patients live with a fistula (4.2%) and 1 was amputated (2.1%).

**Conclusion:**

Although burdened by a big complication rate, knee arthrodesis with an intramedullary nail provides satisfactory results and is a good alternative to above-knee-amputation. The Wichita^®^ fusion nail shows a tendency to better results compared to the two other nails.

**Level of evidence:**

Case series, level IV

## Introduction

Widely used until the beginning of the twentieth century, knee arthrodesis (KA) is considered nowadays to be a salvage procedure, due to the progress in complex revision total knee arthroplasty (TKA) surgery. The goal of KA is to provide an indolent stable limb and is an alternative to an above-knee amputation [13, 23, 24]. It may provide a superior functional outcome and ambulatory status compared with above-the-knee amputation [6, 13, 18]. The indications for KA are severe bone loss, chronic infection, compromised soft tissues and extensor mechanism deficiencies. Up to 93% of the time, this is due to failed TKA [11]. Available techniques for KA include compression plating, external fixation and intramedullary nailing (IMN).

The overall fusion rate is 68–95% [1, 3, 7, 15]. Many authors have demonstrated the superiority of the IMN for KA compared to external fixation regarding the consolidation rate [1, 7, 8, 11, 15, 16, 18]. Patients poorly tolerated external fixators because it is bulky and requires a non-weight bearing period. They also frequently present with pin track infections. Long IMN enables dynamic compression during walking promoting fusion but makes the dissemination of germs in the diaphysis of the two bones possible [5, 19, 21]. The overall complication rate of KA is 33–57% [2, 7, 11, 19], depending on the technique. These include new or persistent infection, peri-implant or implant fracture, nonunion, deep venous thrombosis and neuro-vascular lesions.

**Fig. 1:**
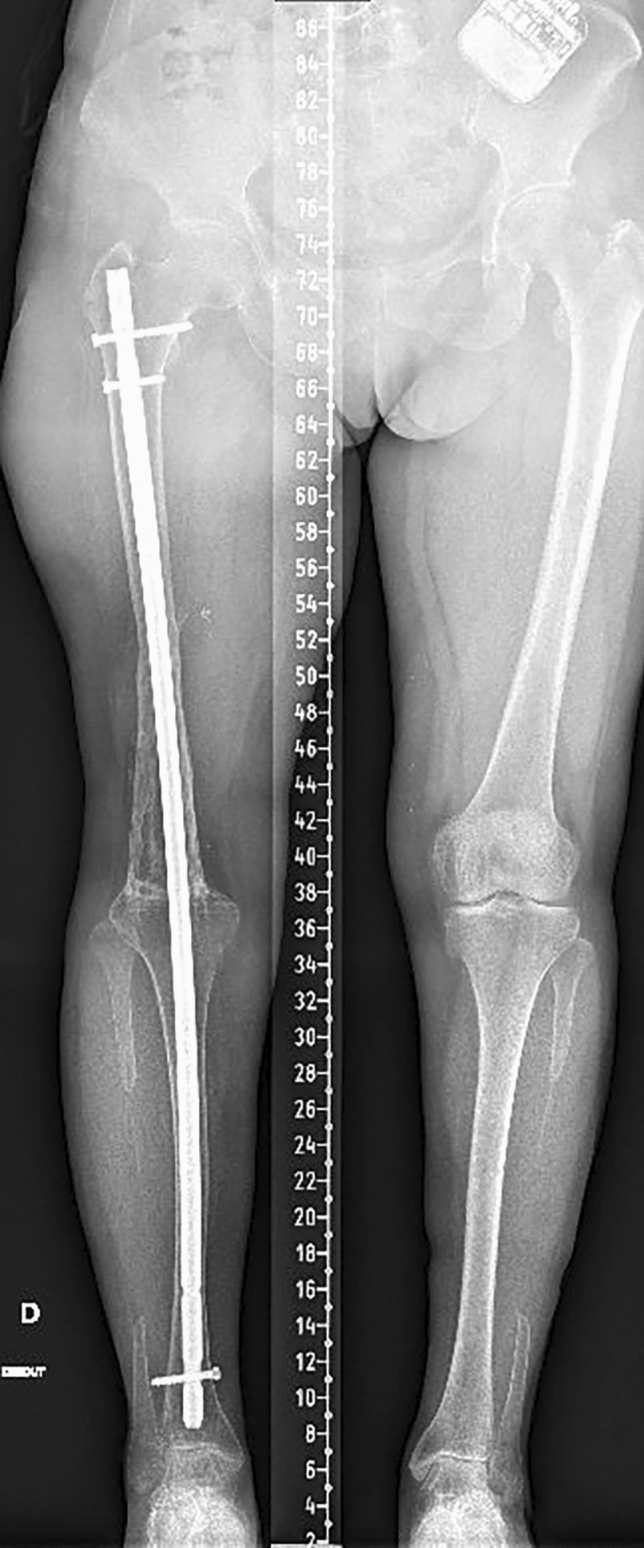
Standing whole leg radiograph of KA using T2AN at 1 year

**Fig. 2:**
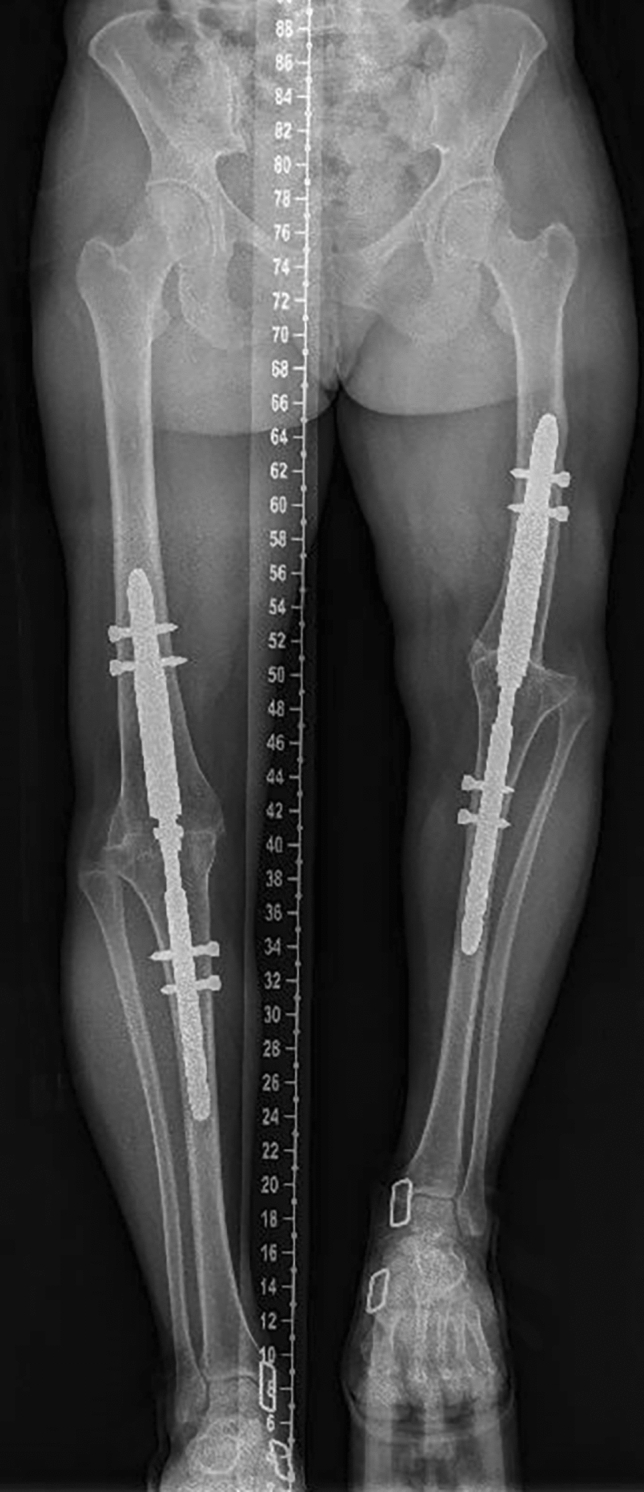
Standing whole leg radiograph of bilateral KA using WFN at 1 year

**Fig. 3:**
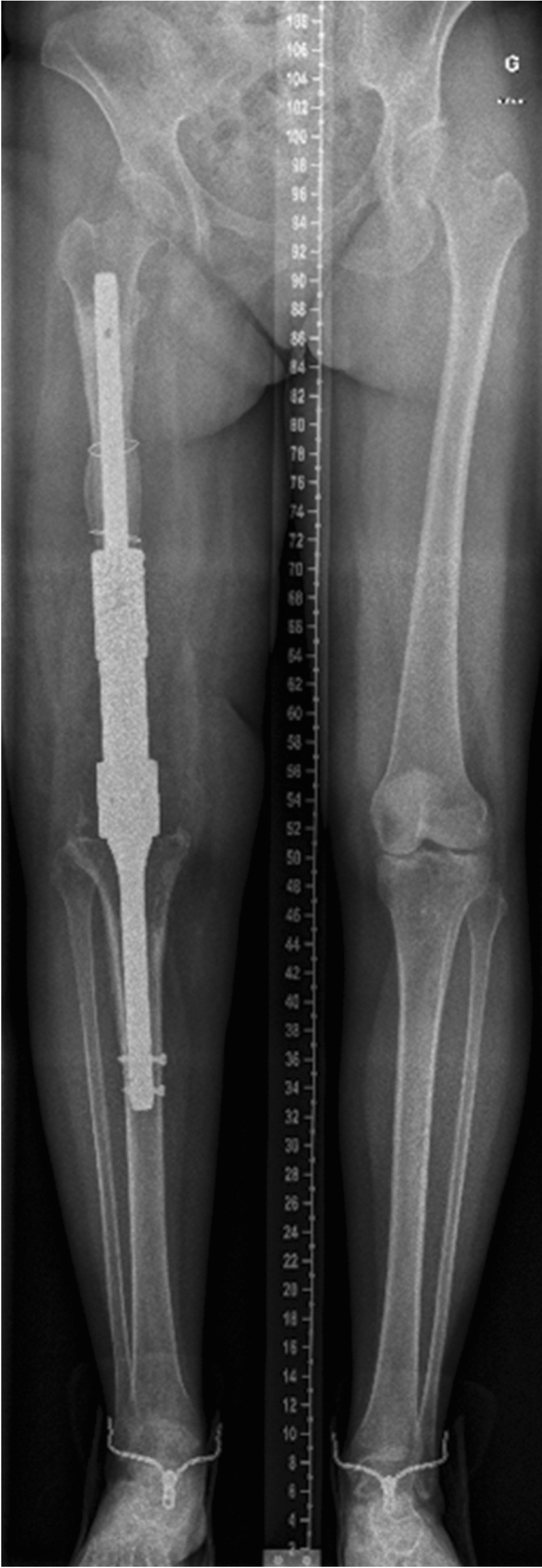
Standing whole leg radiograph of KA using OKA at 1 year

The department is part of a university hospital trauma level 1 centre. IMN was mostly used for KA. Different nails are available. The T2^™^ Arthrodesis Nail (T2AN; Stryker, Kalamazoo, MI, USA) (Fig. 1) is introduced through the greater trochanter and the surgeon does not need to access the knee. This is advantageous in cases of fragile soft tissues. However, it has an elasticity due to its length that does not allow a good compression at the site of arthrodesis. Moreover, its large working length increases the risk of fracture. The Wichita^®^ fusion nail (WFN; Stryker Orthopedics, Mahwah, NJ, USA) (Fig. 2) is a modular short nail. It respects the principles of Charnley for a good fusion: rigid fixation, good osseous contact and compression [4, 5]. Besides, only the knee needs to be approached and the ideal position for the arthrodesis is easily controlled. In case of great bone defect, which would induce an important limb shortening, the OsteoBridge® Knee Arthrodesis (OKA; Merete medical, Berlin, Germany) (Fig. 3) can be a valid option. This nail does not require bony fusion but the constraints at its anchor points are high and the fatigue of the implant may lead to fracture.

The hypothesis of the study is that WFN has, due to the above listed arguments, a better fusion rate. Therefore, the clinical and radiological results of the KA carried out in the department between 2000 and 2018 by IMN were assessed and the different nails used were compared. The primary outcome is the fusion rate for KA performed by a WFN versus a T2NA. The secondary outcome is the complication rate and the third outcome is to determine if there is any factor affecting the clinical outcome.

## Methods

### Data sources

This mono-centric, retrospective case series study included all adult patients undergoing KA with IMN between 01.01.2000 and 07.01.2018, performed by a single senior surgeon in the department, based on the hospital records. Additional inclusion criteria were: minimum follow-up of 12 months, a sufficient patient dataset and complete radiographic imaging studies. Patients lost to follow-up and KA not using an IMN were excluded.

For outcome evaluation, all available data and imaging from follow-up visits in the outpatient clinic were used. Clinical and demographical data included age (years old = y.o.) at the KA, gender, height, Body Mass Index [BMI: kilograms (kg)/metre (m)^2^], length of hospital stay, blood loss, duration of surgery or operative time, pre-operative haemoglobin, number of previous surgeries, indication for KA, Anderson Orthopaedic Research Institute (AORI) classification for bone deficiency [9], fusion rate, time to fusion, complications and function outcome using the Parker score and VAS at the last follow-up [18]. Radiographic fusion was documented based on three or more bridged cortices on plain X-rays [22] and judged by an independent reviewer not associated with the surgical procedures.

### Population

A total of 53 KA performed between 2000 and 2018 were identified. Five cases were excluded: in three cases, plating had been used and two cases presented incomplete clinical records. A total of 15 T2 AN, 6 OKA and 27 WFN were implanted. The mean clinic and radiological follow-up was 9.8 ± 3.8 years (2.6–18 years). The groups were comparable in age, gender and BMI (Table 1).

**Table 1:** Population characteristics

	All	T2 AN	OKA	WFN
Age (y.o.)	63.6 ± 14 (22–85)	64.7 ± 17 (22–85)	64.3 ± 8 (49–72)	61.3 ± 11 (38–83)
Gender F:M	23:25	13:14	3:3	7:8
BMI (kg/m^2^)	28.4 ± 6.8 (20–51)	28.3 ± 8 (20–51)	30 ± 7 (25–45)	26.2 ± 4 (20–32)

### Choice of implant

The WFN was the preferred implant. The T2AN was used when the WFN was not available, before 2004 and after 2015, or when a diaphyseal fracture needed to be bridged above or below the KA. The T2AN nail was also used for one case with a fragile vascular bypass around the knee. The OKA was the implant of choice when major bone defects would have led to a limb shortening of more than 5 cm.

### Data collection and statistics

Statistical analysis of the data was carried out using IBM SPSS Statistics for Windows, Version 24.0 (Armonk, NY: IBM Corp). Measurement accuracy is assumed at one decimal. Kaplan–Meier survival analysis with asymmetrical 95% confidence intervals was used to assess implant survival. The different groups were compared using the ANOVA and Chi^2^ test and the significance of different factors were assessed using the Pearson correlation coefficient test, with a significance assumed at *p *< 0.05. The study population was analysed globally and depending on the type of IMN used.

## Results

Fusion occurred in 89.6% of the cases with a trend of better consolidation for the WFN, 92.6% against 80% for the T2AN (*p* = n.s.). Time to consolidation was 6.9 ± 3.1 months. The latter tends to be shorter with the WFN compared to T2AN (*p* = n.s.) (Table 2). Two predictive factors were isolated for the fusion rate. The first was the number of previous surgeries before KA. Patients who underwent more than 9 previous surgeries had a lower fusion rate (*p* = 0.05) and more than 5 for WFN alone (*p* < 0.01). The second was a bone stock of 3 before the surgery (*p* > 0.01). There was no radiologic loosening in the OKA group at the last follow-up.

**Table 2:** Primary and secondary outcomes

	All	T2 AN	OKA	WFN
Primary outcomes				
Fusion rate % (cases)	89.6% (43)	80% (12)		92.6% (24)
Fusion time (months)	6.9 ± 3.1 (3–19)	8.1 ± 4.3 (5–19)		6.3 ± 2.2 (3–12)
Secondary outcomes				
Parker score	6.9 ± 2.8 (0–9)	6.5 ± 2.8 (1–9)	5.3 ± 2.7 (1–9)	7.4 ± 2.7 (0–9)
VAS	1.9 ± 2 (0–8)	1.3 ± 1.7 (0–6)	2.8 ± 0.8 (2–4)	2.7 ± 2.4 (0–8)
Bone stock	2.1 ± 1 (0–3)	2.2 ± 0.8 (1–3)	3 ± 0	1.9 ± 1.2 (0–3)
Previous surgeries (number)	90% (43)	100% (15)	100% (6)	81.5% (22)
Number of previous surgeries	4.1 ± 2.9 (0–12)	4 ± 2.5 (1–12)	6.3 ± 3.2 (3–11)	3.7 ± 2.9 (0–11)

The clinical outcome measured using the Parker score shows an overall result of 6.9 ± 2.8 (0–9) and was higher for the WFN group when compared to the OKA and the T2AN groups (*p* = n.s.) (Table 2). Two predictor factors were isolated. The fusion was a predictive factor of good outcome (OKA excepted) *p* > 0.01. The height of the patient, above 179 cm, was a factor associated with a good Parker score (*p* = 0.02). The pain measured by the VAS shows a global result of 1.9 ± 2 (0–9) and was higher for the WFN group when compared to the OKA and the T2AN groups (*p* = 0.03) (Table 2). There was no difference between the OKA and T2AN groups (*p* = n.s.).

Most patients underwent previous surgeries on the affected knee prior to KA. The mean number of surgical procedures between the primary surgery and the KA was higher for the OKA group compared to the WFN and the T2AN groups. The remaining few underwent KA as primary knee surgery, due to congenital knee dislocations with an important length inequality between the segments, inflammatory disease with a major fixed flexum, chronic multiple joint infections with a complement deficit which imply a high risk of TKA infection and trauma associating major bone loss and ligamentous instability.

**Fig. 4:**
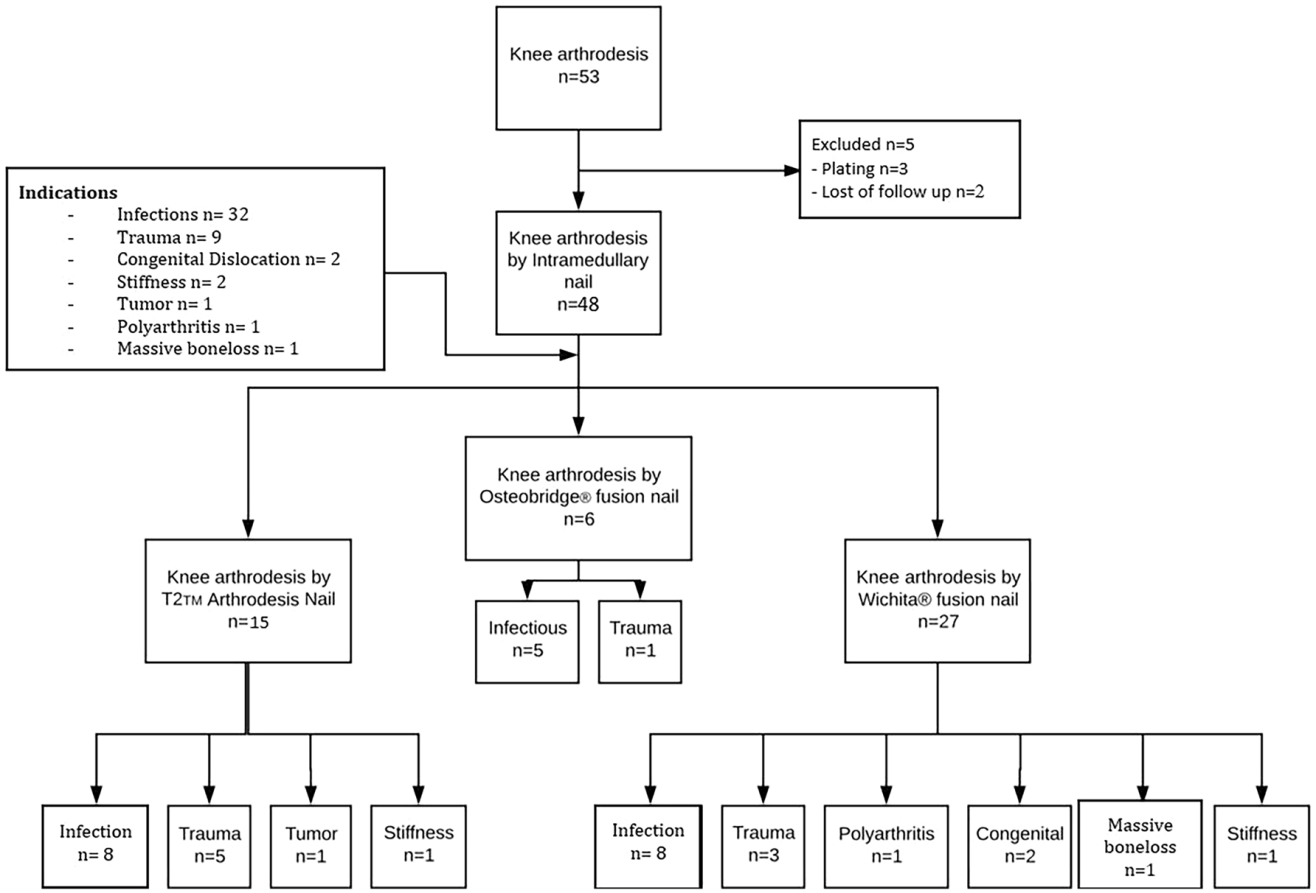
Flow chart of KA and indications

The reason for KA was infection in 66.7% of cases (30 TKA and 2 native knees) and trauma in 20.8% of cases (10 cases). Only 12.5% (6 knees) of the operations were performed for other conditions (Fig. 4). Of the 30 failed TKA (62.5% of KA), only 4 (13.3%) were fused in a one-stage surgery, two with a WFN and two with the T2 AN. The 26 (86.7%) others required a two-stage surgery. The indications for a KA instead of a TKA revision are listed in Table 3. The 6 patients treated with OAK had severe bone loss in addition to another condition.

**Table 3:** Diagnose and indication for knee arthrodesis

Diagnose	Number of knees
Chronic infection after TKA	30 (62.5%)
Chronic native knee infection	2 (4.2%)
Traumatic lesion around the knee	10 (20.9%)
Congenital knee dislocation	2 (4.2%)
Severe arthrosis with ankylosis	2 (4.2%)
Tumour	1 (2.1%)
Polyarthritis with fixed flexum	1 (2.1%)
Indication	
Extensor mechanism deficiency	24 (50%)
High risk of infection regarding comorbidities	9 (18.8%)
Compromised soft tissues	8 (16.7%)
Severe bone loss	5 (10.4%)
Ankylosis	2 (4.2%)

Blood loss [921 ± 562 ml (50–2500)], duration of surgery [144.7 ± 47.7 min (60–280)] and pre-operative haemoglobin [113.9 ± 22.4 g/l (70–180)] were comparable amongst the groups.

### Complications

The rate of early medical complications was 37.5% (Table 4), out of which 10.4% had an early complication within 30 days after the surgery, requiring revision surgery. Two patients had an early infection and needed surgical debridement and antibiotics. One patient had a lesion of the peroneal nerve treated by a delayed tendon transposition.

**Table 4:** Complications within 30 days

Medical complications	T2AN	OKA	WFN
Anaemia	3 (20%)	3 (50%)	5 (18.5%)
Thromboembolic event	2 (13.3%)	1 (16.7%)	1 (3.7%)
Heart failure	0	1 (16.7%)	0
Pneumonia	1 (6.7%)	0	1 (3.7%)
Early complication requiring revision surgery			
Hematoma	0	0	1 (3.7%)
Wound complication	0	1 (16.7%)	1 (3.7%)
Neurological lesion	1 (6.7%)	0	0
Early infection	1 (6.7%)	0	1 (3.7%)
Total early complication rate	40% (6/15)	83.3% (5/6)	34.6% (9/27)

The late complications rate, requiring a revision surgery, was 20.8% and are summarised in Table 5. The rate was 50% (3/6 cases) of the OKA, 17% of the T2AN and 15% of the WFN. The two patients who had persistent infections had both medical comorbidities that could have negatively affected the outcome. One showed lack of compliance in the context of chronic alcoholism. Both were offered revision surgery but declined, and preferred to live with a fistula. Antibiotics were stopped after fistulation.

**Table 5:** Late complication requiring surgical revision

Nonunion 10.4%	Indication KA	Previous surgeries	Implant	Failure reason	Revision surgery
Case 1	Failure infected TKA	3	T2AN + free skin flap	Lack of compression	KA by WFN
Case 2	Failure infected TKA	6	WFN	Fracture of implant	KA by WFN + plates
Case 3	Failure infected TKA	4	WFN	Poor bone stock	KA by plates
					Revision by T2AN two stage
Case 4	Failure infected TKA	6	WFN + free skin flap	Lack of compression	KA by plates
Case 5	Failure infected TKA	12	OKA	Poor bone stock	KA by T2AN + cement

Infection 4.2%	Indication KA	Previous surgeries	Implant	Comorbidities	Bacteria	Revision surgery
Case A	Failure infected TKA *S. Lugdunensis, Actinomyces Neuii*	5	T2AN + Skin flap	/	*P. Aeruginosa*	KA by T2AN one stage
Case B	Failure infected TKA *S. Epidermidis*	6	OKA + free skin flap	Rhythmic and hypertensive heart disease, Obesity	*P. Aeruginosa C. Albicans*	Above knee amputation

Persistent infection 4.2%	Indication KA	Previous surgeries	Implant	Comorbidities	Bacteria	Revision surgery
Case C	Infection of tibial plateau osteosynthesis *S. Aureus*	5	T2AN	Smoking, alcoholism	*S. Aureus*	Persistent infection with fistula
Case D	Infected peri-TKA osteosynthesis *S. Caprae*	4	OKA + skin flap	Morbid obesity/diabetes non-insulin-dependent	*S. Caprae*	Persistent infection with fistula

Suspicion of infection 2%	Indication KA	Previous surgeries	Implant	Comorbidities	Bacteria	Revision surgery
Case E	Failure infected TKA *S. Epidermidis*	4	WFN	COPD, Sjögren syndrome	/	KA by plates

## Discussion

The most important finding of the present study was a primary fusion rate of 89.6% with time to consolidation being 6.9 months, which is comparable to other studies [7, 11, 12, 14, 17, 20, 22]. Two expected predictive factors for the fusion rate were isolated. The first is having more than 9 previous surgeries and more than 5 for WFN only. KA is commonly perceived as an unfavourable option by most surgeons and patients. Therefore, it is often delayed or discarded. The issue surrounding the timing of knee arthrodesis in such patients is that by the time the patient and the surgeon capitulate to the idea of arthrodesis, the multiple revision surgeries have jeopardised the soft tissues and bone stock [18]. The second predictive factor is a lower bone stock before the KA. This might be explained by the lack of bony contact in defects AORI grade 3, which leads to delayed or nonunion.

The Parker score shows an overall result of 6.9/9 points. Three predictive factors were isolated. First, the fusion was expected since a fused KA provides a pain free and functional limb. The second, a lower VAS, was also an expected result as lower pain allowed for better ambulation, and therefore, a better autonomy. However, a body height of more than 179 cm being a factor associated with a better Parker score was unexpected. Some experts recommend considering amputation above KA in tall (> 185 cm) patients due to bulkiness of a long stiff limb that would cause limitation by sitting in narrow spaces [10]. On average, the men in the study were taller than the women, which is similar to the general population (176.2 ± 8.9 cm for men and 164.4 ± 9.2 cm of the study), one might assume that gender and size could be considered as confounding factors. The gender is not correlated to the clinical outcome with a *p* = n.s. and a linear regression analysis of the Parker score and height corrected by the sex shows a *p* = 0.06. This means that sex is not considered as a confounding factor. The VAS shows a good global result of 1.9/10 with a better result for WFN compared to the other nails.

KA has a high complication rate with up to 57% of patients requiring revision surgery [21] and 14% requiring amputation [11]. This might be explained by the fragility of the multi-operated population. In the series, 37.5% of the patients had an early medical complication, 10.4% of the patients required an early revision surgery and 20.8% of the patients had complications that required a new KA for either nonunion or infection. These numbers are comparable to the literature [1]. Nonunion is related to either poor bone quality or lack of compression. One amputation was reported, representing 2% of the cases.

WFN shows a trend to better results in terms of consolidation, time to consolidation and clinical outcome measured by Parker score and VAS. However, these data were not significantly significant, most likely due to the small sample size. Unfortunately, the WFN was withdrawn from the market in 2016. As the CE marking expired, Stryker Orthopedics could not find a surgical partner to produce an update. One of the reasons is the fact that this surgery has become rare.

To the best of our knowledge, this is the second biggest cohort of KA by IMN after Hungerer et al with 81 cases [14]. It has one of the longest follow-up with 9.8 years. A single senior surgeon performed all the surgeries. The three groups are comparable to each other and to the literature [7, 11, 12, 15, 17, 20, 22]. Through this study, the Charnley principles were confirmed [4, 5], showing better results with a modular nail that provides a stable fixation, an intra-operative compression across the bone surfaces and dynamic compression during mobilisation. A better bone stock allows a better contact surface, and therefore, a better fusion rate. Thus, the timing to KA is a key point to fusion and a good clinical outcome. In addition, the nail is implanted through a single incision. Thus, it can be implanted in patients with an ipsilateral THA [15, 23].

This study presents its limitations. It is a retrospective study with a small number of patients. The groups have variable number of patients. This might explain the lack of statistical significance of some results. The fusion of the KA was only judged by X-rays, based on the fusion of at least three cortices. This can be questionable due to the lower sensitivity compared to CT scan. However, no routine CT scan was performed as a follow-up tool in the department. Moreover, the number of cortices bridges by callus is the most reliable indicator of fusion and the most objective measure [23]. There is currently no score in the literature validated for KA. All the scores used were adapted from different knee scores due to the lack of knee bending. The aim of KA is to provide an indolent and stable limb to allow the patient to be autonomous. Parker’s score has been validated for another purpose and might be appropriate to measure clinical outcome, as it still gives information about the patient’s autonomy, in and out the house, and their ability to walk. Finally, to assess the indolence of the limb, a Virtual Analogic Scale has been evaluated at the last follow-up.

## Conclusion

These data show that KA with an IMN provides satisfactory results with an acceptable complication rate. Unfortunately, to the best of our knowledge, there is actually no compressive modular knee fusion nail available on the market. Since the study shows its tendency to better results in term of consolidation, time to consolidation and clinical score, this needs to be addressed.
